# Glutamate carboxypeptidase activity in human skin biopsies as a pharmacodynamic marker for clinical studies

**DOI:** 10.1186/1479-5876-9-27

**Published:** 2011-03-09

**Authors:** Camilo Rojas, Marigo Stathis, Michael Polydefkis, Michelle A Rudek, Ming Zhao, Gigi J Ebenezer, Barbara S Slusher

**Affiliations:** 1 Brain Science Institute, Johns Hopkins School of Medicine, 855 North Wolfe Street, Baltimore, MD 21205, USA; 2 Department of Neurology, Johns Hopkins School of Medicine, 1550 Orleans Street, Baltimore, MD 21231, USA; 3 Department of Oncology, Johns Hopkins School of Medicine, 1650 Orleans Street, Baltimore, MD 21231, USA

## Abstract

**Background:**

Glutamate excitotoxicity is thought to be involved in the pathogenesis of neurodegenerative disease. One potential source of glutamate is N-acetyl-aspartyl-glutamate (NAAG) which is hydrolyzed to glutamate and N-acetyl-aspartate (NAA) in a reaction catalyzed by glutamate carboxypeptidase (GCP). As a result, GCP inhibition is thought to be beneficial for the treatment of neurodegenerative diseases where excess glutamate is presumed pathogenic. Both pharmacological and genetic inhibition of GCP has shown therapeutic utility in preclinical models and this has led to GCP inhibitors being pursued for the treatment of nervous system disorders in human clinical trials. Specifically, GCP inhibitors are currently being developed for peripheral neuropathy and neuropathic pain. The purpose of this study was to develop a pharmacodynamic (PD) marker assay to use in clinical development. The PD marker will determine the effect of GCP inhibitors on GCP enzymatic activity in human skin as measure of inhibition in peripheral nerve and help predict drug doses required to elicit pharmacologic responses.

**Methods:**

GCP activity was first characterized in both human skin and rat paw pads. GCP activity was then monitored in both rodent paw pads and sciatic nerve from the same animals following peripheral administration of various doses of GCP inhibitor. Significant differences among measurements were determined using two-tailed distribution, equal variance student's t test.

**Results:**

We describe for the first time, a direct and quantifiable assay to evaluate GCP enzymatic activity in human skin biopsy samples. In addition, we show that GCP activity in skin is responsive to pharmacological manipulation; GCP activity in rodent paws was inhibited in a dose response manner following peripheral administration of a potent and selective GCP inhibitor. Inhibition of GCP activity in rat paw pads was shown to correlate to inhibition of GCP activity in peripheral nerve.

**Conclusion:**

Monitoring GCP activity in human skin after administration of GCP inhibitors could be readily used as PD marker in the clinical development of GCP inhibitors. Enzymatic activity provides a simple and direct measurement of GCP activity from tissue samples easily assessable in human subjects.

## Background

Excess glutamate has been shown to be neurotoxic in many degenerative diseases of the central and peripheral nervous system [1]. One potential source of glutamate is N-acetyl-aspartyl-glutamate (NAAG), a dipeptide found in the brain and peripheral nerves [2]. Glutamate carboxypeptidase (GCP) catalyzes the hydrolysis of NAAG to glutamate and N-acetyl-aspartate (NAA) [3]. There are two known GCP enzymes in the nervous system with similar pharmacological profiles: GCPII and GCPIII. GCPII, the more widely studied homolog, exhibits a high level of expression and it is found on the cell surface of astrocytes and non-myelinating Schwann cells [4-6]. GCPIII message on the other hand, is expressed in mouse cortical and cerebellar neurons in culture [7]. Inhibition of the GCP-catalyzed reaction should be beneficial for the treatment of degenerative diseases associated with excess glutamate. In fact, both genetic and pharmacological inhibition of GCP has been found to be neuroprotective in a variety of cell and animal models of disease involving excess glutamate [8-17]. Based on these data, GCP inhibitors are currently being pursued in the clinic as therapeutics for the treatment of peripheral neuropathy and neuropathic pain [18].

Clinical development of a drug can be aided by pharmacodynamic (PD) marker assays to predict drug doses required to elicit pharmacologic responses. Until recently, monitoring NAAG levels in biological matrices (e.g. CSF, plasma, and urine) was considered the PD marker of choice to monitor GCP inhibition [19]. For clinical studies, the best biological matrix to evaluate CNS/PNS penetration is cerebrospinal fluid. However, sample collection requires considerable skill and it is uncomfortable to patients. In addition, NAAG measurements involve the use of HPLC or LC-MS/MS [19] and are only a surrogate marker of enzyme inhibition. Quantifying GCP enzymatic activity on the other hand, provides a direct measurement for monitoring enzyme inhibition and is relatively straightforward to carry out. Until recently, GCP activity measurements were thought to be unfeasible as PD marker assays in the clinic because GCP was thought to be present only in nervous tissue, prostate, intestinal tract, and kidney, tissues that are not easily accessible for collection during clinical studies [20]. However, local administration of GCP inhibitors have been shown to be analgesic in peripheral pain in rats [21] and NAAG is known to be synthesized and localized in spinal sensory ganglia [22]. Further, GCP is located in Schwann cells [4,5] which exist in the epidermis [23]. Consequently, we set out to determine if GCP was measureable in human skin. In this report, we describe for the first time, quantifiable GCP activity in human skin biopsy samples. Further, to determine if GCP activity in skin is amenable to pharmacological manipulation, we conducted rodent studies on GCP activity in rat paws after dosing with GCP inhibitor. We report robust GCP activity in rodent paws which is sensitive to inhibition in a dose response manner following peripheral administration of a GCP inhibitor. Further, inhibition of GCP activity in rodent paws was shown to correlate to GCP inhibition in peripheral nerve.

## Methods

### Human skin biopsy collection

Punch skin biopsies (3 mm) were obtained from the distal thigh of healthy volunteers after anesthesia with 0.5 cc 2% lidocaine subcutaneous injection [24]. The protocol was approved by the Johns Hopkins Institutional Review Board in compliance with the Helsinki declaration. Samples were placed in cold Tris buffer (pH 7.4) and GCP enzymatic activity was carried out within 1 h of collection.

### Rodent drug dosing and paw and sciatic nerve sample collection

All experimental protocols were approved by the Institutional Animal Care and Use Committee of SoBran, Inc., Baltimore and adhered to all of the applicable institutional and governmental guidelines for the humane treatment of laboratory animals. Rats (male Wistar) were administered vehicle (HEPES saline, pH 7, 50 mM) or 2-PMPA (1, 10 and 100 mg/kg, i.p.) using a dosing volume of 2 mL/kg. There were 10 animals in each group. Animals were sacrificed 1 h after 2-PMPA or vehicle administration. 2-PMPA brain concentrations were previously shown to be highest 50 - 75 min after i.p. administration [12]. Skin was collected from the planter hindpaw by 3 mm skin biopsy dissection and stored at -80°C until ready for analysis. In order to obtain sciatic nerve, 1-2 cm incisions were made on the skin on top of the mid thigh so that sciatic nerve, gluteus superficialis muscle and biceps femoris muscle became exposed. The three were then separated and 5 mm of sciatic nerve was dissected out.

### Human skin biopsy and rodent paw and sciatic nerve sample preparation

Human skin biopsies were sonicated in Tris buffer (pH 7.4, 40 mM, 0.5 mL) for 1 min in ice. The mixture was centrifuged for 2 min at 16000 × g; the supernantant (containing cytosolic fraction) was removed and the resulting pellet (containing plasma membrane) was reconstituted in 70 μL assay buffer (Tris pH 7.4, 40 mM containing 1 mM CoCl_2_) and used as source of GCP in the activity assay. Rat paw pads and sciatic nerve isolated from vehicle and 2-PMPA treated animals were sonicated for 2 min in ice. The mixture was centrifuged for 2 min at 16000 × g and the resulting pellet was reconstituted similar to the pellets obtained from the human skin dissections.

### Measurement of GCP activity in human skin biopsies and rodent paw pads

GCP activity measurements were carried out following published procedures [3,25]. Briefly, the reaction mixture contained [^3^H]-NAAG (70 nM, 50 Ci/mmol) and reconstituted pellet (human skin, paw pad, or sciatic nerve) in Tris-HCl containing 1 mM CoCl_2 _in a total volume of 90 μL. The reaction was carried out at 37°C at different times as indicated, and stopped with ice-cold sodium phosphate buffer (pH 7.4, 0.1 M, 90 μL). When human skin was used as GCP source, the reaction was carried out in the presence and absence of the selective GCP inhibitor 2-PMPA (1 μM). When rat tissue was used from the *ex vivo *study, 2-PMPA was administered i.p. and the animals were sacrificed and their paw pads removed for GCP enzymatic determinations. In both cases, blanks were obtained by incubating the reaction mixture without pellet. Duplicate aliquots of 90 μL from each terminated reaction was transferred to a well in a 96-well spin column containing AG1X8 ion- exchange resin; the plate was centrifuged at 1000 rpm for 5 minutes using a Beckman GS-6R centrifuge equipped with a PTS-2000 rotor. [^3^H]-NAAG bound to the resin and [^3^H]-glutamate eluted in the flow through. Columns were then washed twice with formate (1 M, 90 μL) to ensure complete elution of [^3^H]-glutamate. The flow through and the washes were collected in a deep 96-well block; from each well with a total volume of 270 μL, a 200 μL aliquot was transferred to a glass scintillation vial, to which 10 ml of Ultima-Gold (Perkin Elmer) was added. The radioactivity in each vial corresponding to [^3^H]-glutamate was determined via a Beckman LS-6000IC scintillation counter. Radioactivity values in dpm were converted to fmoles of glutamate using the relation 1 pCi/2.2 dpm and the specific activity of [^3^H]-glutamate (same as that of [^3^H]-NAAG: 1 fmole/50 pCi). As a result, if 16711 dpm [^3^H]-glutamate were measured after incubating 10 mg tissue for 1 h, the normalized activity would be: 16711 dpm × (1 pCi/2.2 dpm) × (1 fmole/50 pCi)/10 mg tissue = 15 fmole/h/mg tissue.

### Statistical Analysis

Significant differences among measurements were determined using two-tailed distribution, equal variance student's t test.

### Determination of 2-PMPA concentration in rodent paws by LC-MS/MS

Frozen samples were thawed in a water bath at ambient temperature and subjected to a liquid extraction using MeOH. Samples were placed in brown glass vials containing 500 μL of 100% MeOH. The vial was capped and mixed vigorously for 10 sec on a vortex-mixer followed by 30 min on an automated multitude shaker, followed by incubation for 24 h at 4°C. The top organic layer was transferred to a disposable borosilicate glass culture tube (13 × 100 mm) and evaporated to dryness at 40°C under a gentle stream of nitrogen. The residue was reconstituted in 100 μL acetonitrile-water (1:1, v/v) containing the internal standard, temazepam (50 μg/mL), by vortex mixing (30 sec) and immersion in an ultrasound bath (5 min). The sample was transferred to a 250 μL polypropylene auto sampler vial sealed with a Teflon crimp cap, and a volume of 50 μL was injected onto the HPLC instrument for quantitative analysis using a temperature-controlled auto sampling device operating at 10°C.

Chromatographic analysis was performed using a Waters ACQUITY UPLC (Milford, MA, USA). Separation of the analytes from potentially interfering material was achieved at ambient temperature using a Waters Altantis column (100 × 2.1 mm i.d.) packed with a 3 μm ODS stationary phase, protected by a guard column packed with 3.5 μm RP18 material (Milford, MA, USA). The mobile phase used for the chromatographic separation was composed of acetonitrile-water (60:40, v/v) containing 0.1% formic acid, and was delivered isocratically at a flow rate of 0.3 mL/min. The column effluent was monitored using an AB SCIEX TRIPLE QUAD 5500 triple-quadrupole mass-spectrometric detector (Applied Biosystems, Foster City, CA, USA). The instrument was equipped with an electrospray interface, operated in a positive mode and controlled by the Analyst version 1.5 software (Applied Biosystems). The spectrometer was programmed to allow the [MH^+^] ion of 2-PMPA at m/z 226.8 and that of the internal standard at m/z 301.1 pass through the first quadrupole (Q1) and into the collision cell (Q2). The daughter ions for 2-PMPA (m/z 191.1) and the internal standard (m/z 255.1) were monitored through the third quadrupole (Q3). Calibration curves were generated over the range of 200 to 10,000 ng/mL. Mouse paw pad samples were then quantitated in μg/g as: nominal concentration (ng/mL) × 0.0625 (standardized dilution) × sample weight (in mg).

## Results and Discussion

### GCP II activity is present in human skin biopsies

Skin biopsies from human volunteers were homogenized, the homogenate was centrifuged and the pellet was used as source of GCP in the enzyme activity assay. Reconstituted pellet was then incubated with [^3^H] NAAG and production of glutamate was determined in the presence and absence of 2-PMPA, a highly selective GCP inhibitor (Methods) [26]. When pellets obtained from human skin biopsy were used, conversion to glutamate was 11 ± 0.2 fmole glutamate generated/h/mg tissue. GCP activity monitoring in human skin was attempted previously, but reported to exist below the limit of detection [27]. In this study, according to previous findings [27], we found that homogenate preparations of human skin exhibited a very low GCP activity that was difficult to measure. However, when using pellet preparations (methods) as source of GCP, we found significant measurable activity in human skin biopsies that was inhibited by 90% when 2-PMPA, a highly specific GCP inhibitor, was added to the assay mixture.

**Figure 1:**
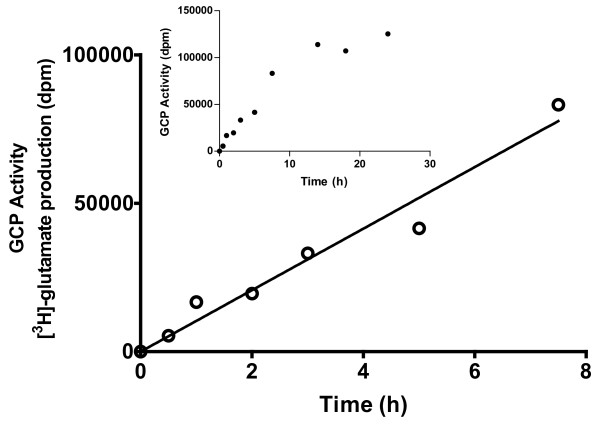
**Dependence of GCP activity in human skin biopsy on time of incubation **- Human skin biopsies were sonicated for 2 min in ice. The resulting mixture was centrifuged at 16000 × g; precipitate from each preparation was used as GCP source in the activity assay. Incubations with [^3^H] NAAG (70 nM) at 37°C were carried out at 0.5, 1, 2, 3, 5, 7.5, 14, 18 and 24 h. Time points correspond to incubations carried out with biopsies obtained from different donors. Major plot illustrates the correspondence of enzyme activity ([^3^H]-glutamate production in dpm) with time while linearity was observed. Inset illustrates GCP activity measured at times up to 24 h.

A time course of glutamate production after different incubation times (0.5, 1, 2, 3, 5, 7.5, 14, 18 and 24 h) was carried out. Due to the limited number of samples that can be obtained from one person at a time, samples from different patients were used in this study. Consequently, each time point was an independent determination; pellets were prepared from separate skin biopsies from different volunteer donors over two separate days. GCP activity was found to be linear for the first 7.5 h of incubation (Figure 1). [^3^H]-NAAG at 70 nM (~770,000 dpm) provided robust sensitivity to follow GCP activity; there were approximately 5,000 and 80,000 dpm of [^3^H]-glutamate after 0.5 and 7.5 h incubation respectively. These values corresponded to 0.6 and 10% conversion of reactant to product indicating that sufficient substrate concentration was used and that if additional GCP activity had been present, additional activity would have been observed. The linear relationship with respect to time using samples from different donors suggests that GCP levels among normal volunteers are relatively similar.

### GCP activity is present in rodent paw pads

A parallel determination of GCP activity was carried out using male Wistar rat paw pads. Reconstituted pellet preparations from rat paw pads were used as source of GCP II and incubated with [^3^H] NAAG. The amount of GCP activity in rat paw pads was found to be 15 ± 0.2 fmole glutamate generated/h/mg tissue). Interestingly, the amount of GCP activity found in rat paw pads (15 ± 0.2 fmole/h/mg tissue) was similar to that obtained from human skin (11 ± 0.2 fmole/h/mg tissue).

### Peripheral administration of 2-PMPA inhibits GCP activity in rat paw pads in a dose response manner

**Figure 2:**
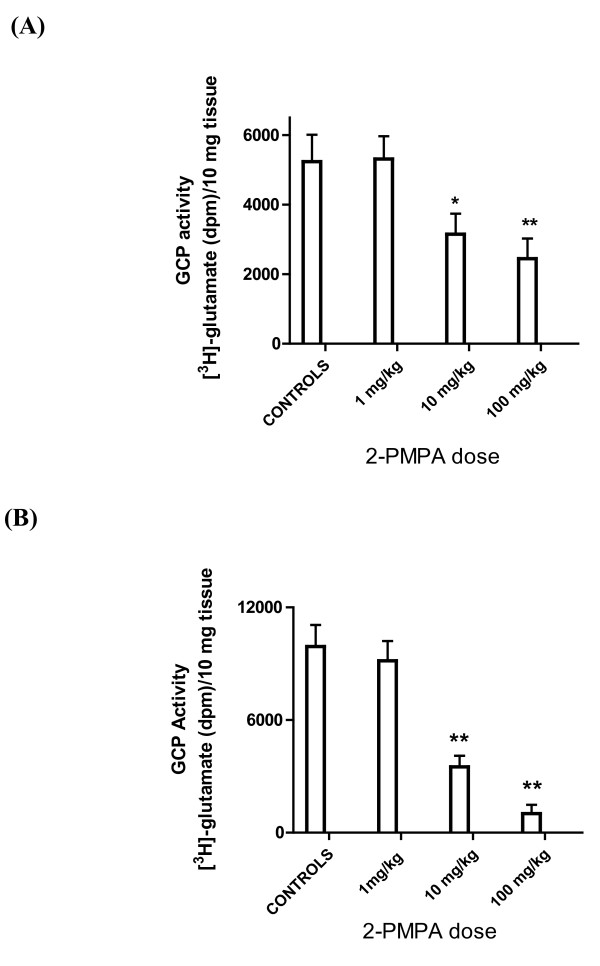
**GCP activity in rat paw pads and sciatic nerve is inhibited by peripheral administration of 2-PMPA **- Rats were treated with 2-PMPA (1, 10 and 100 mg/kg i.p.) as well as vehicle control. Animals were sacrificed 1 h after compound administration, paw pads and sciatic nerve isolated and GCP activity determined (Methods). (A): GCP activity ([^3^H]-glutamate production in dpm/10 mg tissue) in paw pads; * p < 0.05 (B): GCP activity in sciatic nerve. **p < 0.01.

To be useful as clinical PD marker, GCP activity in skin needs to be amenable to inhibition by peripheral administration of GCP inhibitors in a dose response manner. In order to determine if GCP activity in paw pads *in vivo *could be inhibited by peripheral administration of 2-PMPA, rats were treated with 1, 10 and 100 mg/kg 2-PMPA (i.p.) as well as vehicle control. Animals were sacrificed 1 h after compound administration, paw pads isolated and GCP activity determined (Methods). GCP activity in paw pad preparations from animals treated with 1 mg/kg 2-PMPA was similar to that of controls. On the other hand, paw pads from animals treated with 10 and 100 mg/kg exhibited significantly reduced GCP activity: 60 ± 11 and 47 ± 11% respectively when compared to control animals (Figure 2A). Importantly, these are the doses of 2-PMPA previously shown to exhibit therapeutic benefit [13].

### Peripheral administration of 2-PMPA inhibits GCP activity in sciatic nerve in a dose response manner and it correlates to inhibition observed in rat paw pads

Sciatic nerve is the target tissue for GCP inhibitors in clinical trials for peripheral neuropathy and neuropathic pain. Consequently, it is important to demonstrate that there is a correlation of GCP inhibition in skin and peripheral nerve after administration of different doses of GCP inhibitor. GCP activity in sciatic nerve preparations from animals treated with 1, 10 and 100 mg/kg 2-PMPA was reduced to 92 ± 11, 35 ± 6 and 10 ± 4% respectively compared to activity in sciatic nerve isolated from control animals (Figure 2B). Albeit to a different extent, GCP inhibition in sciatic nerve is attained at similar 2-PMPA doses (10 and 100 mg/kg i.p.) as in footpad tissue. Taken together, these results suggested that it will be possible to follow GCP inhibition in the skin as a marker of GCP inhibition in peripheral nerve.

### 2-PMPA is measurable in rat paw pads

**Figure 3:**
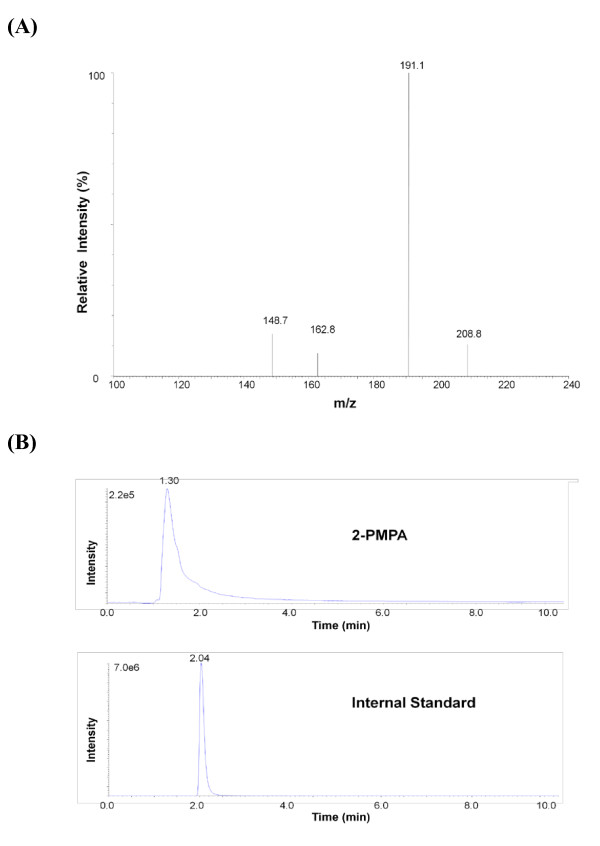
**Measurement of 2-PMPA in rat paw pads using LC-MS/MS **- (A) Daughter-scan product ion spectrum of 2-PMPA. Monitoring was carried out at m/z 226.8 → 191.1 (B) Select rodent paw pad obtained 1 hour after 2-PMPA (100 mg/kg, i.p.) administration. Retention times for 2-PMPA and internal standard (temazepan) were approximately 1.3 and 2.0 min respectively. When rodent paw pads from untreated animals were used, only the internal standard peak was observed.

Given that inhibition of GCP was observed in paw pads, we wanted to confirm the presence of 2-PMPA in paw pads after peripheral administration of 2-PMPA. Animals were given 2-PMPA (100 mg/kg, i.p.), sacrificed 1 h after compound administration and paw pads isolated for direct determination of 2-PMPA levels by LC-MS/MS (Methods). Since 2-PMPA detection by mass spectrometry has low sensitivity due to ion suppression, we chose to analyze samples from animals that had received 100 mg/kg 2-PMPA rather than 10 mg/kg to increase the probability of detecting 2-PMPA. The characteristic fragmentation pattern for 2-PMPA was readily detected (Figure 3A) and the chromatographic peaks of 2-PMPA and internal standard (Figure 3B) allowed for quantitation of material in the sample. Paw pads from animals that were treated with compound showed 38 ± 5 μg/g tissue (n = 9) (Figure 3B) a concentration high enough to inhibit GCP activity [25] while the compound was undetectable in paw pads isolated from vehicle-treated animals.

## Conclusions

As a biomarker of GCP inhibition in the clinic, skin biopsy measurements of GCP activity has three areas of improvement over the prior NAAG bioassay including simpler sample collections, less expensive and time consuming sample analyses, and the ability to quantitate direct vs. indirect measurement of GCP activity. Sample collection for NAAG bioanalysis involves CSF collection which requires considerable skill and can be uncomfortable to patients; the newly described procedure uses skin biopsies which is readily accessible and can be collected multiple times from a single subject permitting the ability to evaluate GCP activity before and after administration of the drug. NAAG analysis uses mass spectrometry which requires a specialized laboratory and expensive instrumentation. The new procedure monitors GCP activity in the skin *ex vivo *by following the conversion of [^3^H]-NAAG to [^3^H] glutamate in a simple enzymatic assay that can be carried out in a standard biochemistry laboratory. Finally, the older procedure involved measurements of NAAG levels as surrogate markers of GCP activity; the new procedure monitors GCP enzymatic activity directly. In short, monitoring of GCP activity in human skin after administration of GCP inhibitors can be readily utilized as a PD marker in the clinical development of GCP inhibitors. The activity assay provides a simple and direct measurement of GCP activity from tissue samples easily assessable in human subjects.

## Abbreviations

GCP: glutamate carboxypeptidase; NAAG: N-acetyl-aspartyl-glutamate; NAA: N-acetyl-aspartate; CSF: cerebrospinal fluid; CNS: central nervous system; PNS: peripheral nervous system; HPLC: High Pressure Liquid Chromatography; LC-MS/MS: liquid chromatography-tandem mass spectrometry; PD: pharmacodynamic; 2-PMPA: 2-(phosphonomethyl) pentanedioic acid.

## Competing interests

CR, MS and BSS are former Eisai employees; Eisai is currently working on the development of a GCP inhibitor.

## Authors' contributions

CR helped with study design and writing of the manuscript. MS carried out GCP activity measurements in the different biological matrices. MP and GJE organized the collection of human skin. MAR and MZ carried out 2-PMPA analysis by LC-MS/MS. BSS conceived the study and study design and guided the writing and editing of the manuscript. All authors read and approved the final manuscript.
